# Dextrocardia With Relapsing Cough in Newly Diagnosed Tuberculosis: A Case Report

**DOI:** 10.7759/cureus.100168

**Published:** 2025-12-27

**Authors:** Chukwuebuka E Ohakpougwu, Emmanuel Ofosu-Brakoh, Wendy M Bebobru, Emmanuel Labram

**Affiliations:** 1 Psychiatry and Behavioral Sciences, Family Health University, Accra, GHA; 2 Psychiatry and Behavioral Sciences, School of Health Sciences Research, Research Institute for Health Science, Chiang Mai, THA; 3 Internal Medicine, School of Medical Sciences, Kwame Nkrumah University of Science and Technology, Kumasi, GHA; 4 Obstetrics and Gynaecology, Lister Hospital and Fertility Centre, Accra, GHA; 5 Anatomical Sciences, Family Health University, Accra, GHA

**Keywords:** antibiotic resistance and behavioral compliance, dextrocardia, kartegener syndrome, primary ciliary dyskinesia (pcd), tuberculosis

## Abstract

Dextrocardia is one of the rarest cardiopulmonary conditions worldwide and has physical health complications such as a risk of repeated upper respiratory tract infections. Our study highlights the incidental diagnosis of pulmonary tuberculosis in a patient not previously known to have dextrocardia and highlights the need for testing for tuberculosis in a case of chronic cough, especially in low- and middle-income countries. Prompt testing also contributes to adequate antimicrobial stewardship and saves lives.

## Introduction

Dextrocardia is a condition in which the apex of the heart is directed toward the right of the mediastinum instead of the left. While it could be an isolated phenomenon, it may also be associated with transposition of the viscera (situs inversus), as seen in conditions such as Kartagener’s syndrome, and congenital heart diseases [1,2]. 

It occurs in about one in 12,000 pregnancies, and as part of the triad of Kartagener’s syndrome (situs inversus, chronic sinusitis, bronchiectasis) in one in 30,000 live births [3-5]. It is also associated with several conditions like impaired ciliary motility, maternal diabetes, and is a common cardiovascular anomaly in conjoined twins [6,7]. Furthermore, impaired ciliary motility, characterised by poor clearance of toxins, may create a suitable medium for bacteria and viruses to thrive, increasing the risk of developing respiratory illness [8]. This could indeed preclude infection by *Mycobacterium tuberculosis*, as due to persistent viral infection, for instance, latent tuberculosis could become activated [9]. 

The condition is diagnosed primarily by radiological means such as plain radiography, ultrasonography, and CT imaging. The diagnosis of pulmonary tuberculosis (PTB) is usually confirmed by nucleic acid amplification, with a clinical background of chronic cough, dyspnea with constitutional symptoms, such as weight loss, fever, anorexia, night sweats, and radiological findings of upper lung lobe consolidation. Upon confirmation, management primarily consists of an initial two-month intensive phase of isoniazid, rifampicin, pyrazinamide, and ethambutol, followed by a four-month course of isoniazid and rifampicin [8].

This case presents the history and management of a patient with dextrocardia and a concurrent diagnosis of PTB. Furthermore, it highlights how lapses in obtaining pertinent clinical investigation can impair antibiotic stewardship and impede positive outcomes in patient management. 

## Case presentation

A 28-year-old female patient, a hotelier and a secondary smoker because of her work, had recurrent episodes of productive cough for one year. She had been to several outpatient departments of different hospitals with the same complaint: productive cough. She was placed on repeated broad-spectrum antibiotics after her recent visits to a healthcare facility over a three-month period and had episodes where her cough appeared to subside and then re-emerged. Unfortunately, a sputum culture had never been done to ascertain causative organisms, and thus, more antibiotics were given, especially amoxicillin-clavulanic acid, as well as antitussives. She presented to our clinic because she was tired of taking antibiotics.

On physical examination, the patient was in no obvious respiratory distress, was not pale, and was anicteric. Vital signs were normal as shown in Table 1.

**Table 1:** Vital signs at first presentation

Vital signs of patient with reference ranges	Temperature (reference range, 36.1-37.2 °C)	Pulse (reference range, 60-100 beats per minute)	Blood pressure (normal, 120/80 mmHg)	Respiratory Rate (reference range, 12-25 cycles per minute)
	36.5	80	130/82	18

**Figure 1:**
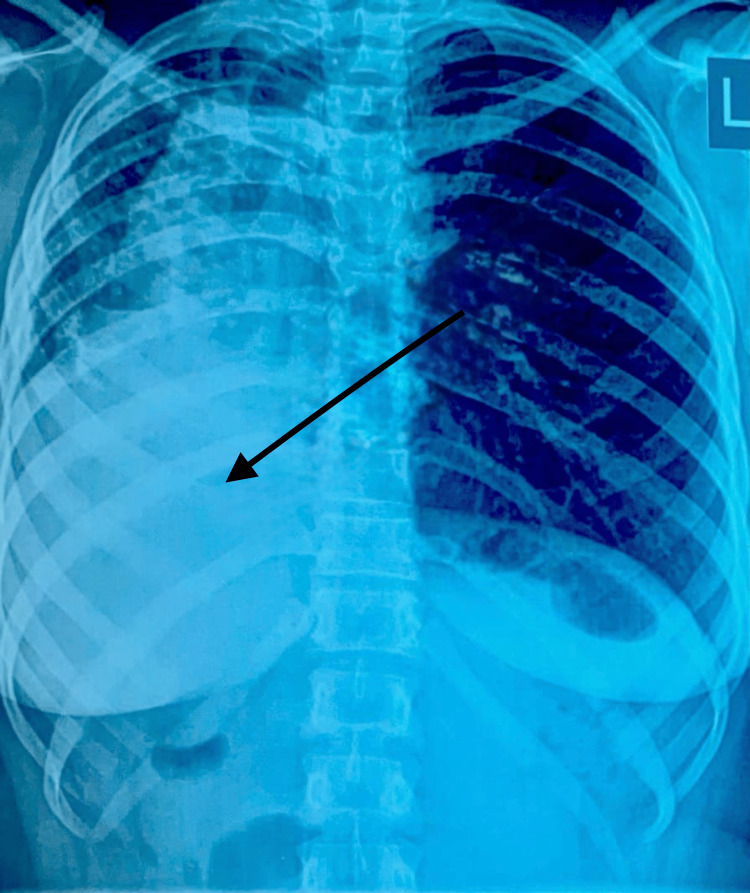
Chest radiograph in posterior-anterior (PA) view Red arrow shows the cardiac silhouette at the right side and opacity in the right lower lung zone with blunting of the costophrenic angle, consistent with dextrocardia.

During the physical exam, the apex beat was absent in the left fifth intercostal space, but on the right side, the apex beat was heard. Air entry was reduced in the right lower lung zones, and percussion notes were dull. Considering the patient's financial circumstances and her chronic use of antibiotics, a Chest X-ray, sputum culture, and GeneXpert test for tuberculosis (Xpert MTB/RIF; Sunnyvale, California, United States) were scheduled even though she did not have any other constitutional symptoms. Initial chest findings were normal. Her chest x-ray was suggestive of dextrocardia, as shown in Figure 1. Hence, we waited for the results of the other lab tests that were ordered.

A week later, her sputum culture was reviewed, and it was positive for only *Staphylococcus aureus*. Furthermore, it was only sensitive to linezolid and clindamycin but resistant to five other antibiotics, as shown in Table 2. This is suggestive of a possible antimicrobial resistance demonstrated by the bacteria's sensitivity to much stronger antibiotics.

**Table 2:** Results of antibiotics sensitivity tests of the Staphylococcus aureus found in sputum culture

Antibiotic	Sensitive	Resistant
Linezolid	Yes	No
Clindamycin	Yes	No
Gentamicin	No	Yes
Chloramphenicol	No	Yes
Vancomycin	No	Yes
Erythromycin	No	Yes
Levofloxacin	No	Yes

Xpert MTB/RIF was positive for *M. tuberculosis* and sensitive to rifampicin, isoniazid, pyrazinamide, and ethambutol, as shown in Table 3. This demonstrated the possibility of a quick resolution of symptoms and improvement of lung health with the right combination of anti-tuberculosis medications. Hence, she was promptly referred to the Infectious Disease Unit of the regional hospital in Ghana, which has a designated tuberculosis treatment program, to initiate treatment immediately. Her treatment regimen consisted of rifampicin, isoniazid, and pyrazinamide.

**Table 3:** Sputum GeneXpert (MTB/RIF assay) results

Parameter	Result
Mycobacterium tuberculosis	Low
Rifampicin resistance	Not detected
Comment	Positive
Recommendations	To start treatment

At the time of writing the report, she had completed the two-month period of the intensive phase of tuberculosis treatment and was currently on the continuation phase of the tuberculosis regimen for the next four months. Her productive cough had largely resolved since her last review, and she has had no adverse events due to the treatment. 

## Discussion

Cilia line various organs from the respiratory tract to the fallopian tube, have a propulsive function, and play an important role in maintaining the left-right axis during embryogenesis [9]. Hence, failure of sensory cilia can lead to randomisation of the left-right axis, leading to situs inversus. When there is a mutation of Dynein genes, which encode motility proteins in respiratory and reproductive tract cilia, there can be inhibition of vital ciliary function [9,10]. Thus, changes in the structure of the ciliary microtubules and uncoordinated ciliary movements caused by the absence of inner or outer or both dynein arms are central to ciliary dysfunction [9]. Indeed, in patients with primary ciliary dyskinesia, there is often a history of bronchitis in infancy with repeated upper respiratory tract infections, making it a diagnostic dilemma, especially if a patient is known to a particular primary care centre for respiratory tract infections over the years. This was certainly the case in this patient and thus accounts in part for why an early diagnosis might not have been made at other centers. 

Mucociliary clearance is an important innate defence that protects the lungs against pathogens and allergens, and when ciliary function is impaired, inhaled particles are unable to move out of the lungs. Signs and symptoms of primary ciliary dyskinesia or immobility include dyspnea, productive cough, bouts of pneumonia, nasal speech, dextrocardia (in some cases), and ciliary beat frequency of less than 10 Hertz [9]. Other objective tests for primary ciliary dyskinesia, such as measurement of ciliary beat frequency using high-speed video microscopy, are impossible in several low- and middle-income countries (LMICs) like Ghana, where the current case was reported from, and this might have also led to missed diagnosis for some decades of life. 

Comorbid tuberculosis infection and dextrocardia have been reported in Nigeria [11], but in Ghana, other case reports have been on dextrocardia presenting with Poland syndrome, intussception, and oesophageal atresia, and as an incidental finding with bowel obstruction [12-15]. Hence, this is the first case report from Ghana on dextrocardia with comorbid tuberculosis to the best of our knowledge. 

The management of cough and flu-like symptoms in primary care settings is contentious, and algorithms for care are not uniform, especially in low-resource settings. Hence, there is an enormous risk of treating cough as a symptom of common cold instead of investigating causative organisms. Hence, further investigations, such as sputum culture and chest X-ray, can help diagnose other life-threatening conditions that may have been missed, such as tuberculosis [16]. Dextrocardia with its comorbid conditions increases patients’ risk of respiratory illness, and work hazards also complicate the condition, as in this patient’s case.

Since the patient was exposed repeatedly to broad-spectrum antibiotics after presenting to various healthcare centers with a chronic cough, this case is a stark reminder of the continued importance of antibiotic stewardship, especially in LMICs, where guidelines might not be strictly adhered to [17]. Hence, there must be continuous effort to encourage physicians to investigate further and explore possible differential diagnoses before treatment.

## Conclusions

Dextrocardia can manifest with respiratory symptoms, and adequate patient evaluation is necessary to help unearth underlying immunocompromising conditions such as tuberculosis. Clinicians must have a high index of suspicion when patients present with chronic relapsing cough and must go the extra mile to uncover possible causes of the presenting symptom to avoid polypharmacy. 
